# Implementing Evidence‐Based Practice in Critical Care Nursing: An Ethnographic Case Study of Knowledge Use

**DOI:** 10.1111/jan.70054

**Published:** 2025-07-04

**Authors:** Jude Ominyi, Ukpai Eze, David Agom, Adewale Alabi, Aaron Nwedu

**Affiliations:** ^1^ School of Nursing, Midwifery & Public Health University of Suffolk Ipswich UK; ^2^ Chester Medical School, Faculty of Health, Medicine and Society University of Chester Chester UK; ^3^ School of Nursing, Midwifery & Health Education University of Bedfordshire Luton UK; ^4^ School of Health & Care Coventry University Coventry UK; ^5^ Department of Nursing Science David Umahi Federal University of Health Sciences Ebony State Enugu State Nigeria

**Keywords:** acute care, critical care nursing, evidence‐based practice, experiential knowledge, focused ethnography, knowledge utilisation, leadership, organisational culture

## Abstract

**Aim:**

To explore how critical care nurses access, negotiate and apply knowledge in high‐pressure clinical environments, focusing on organisational, cultural and leadership factors influencing evidence‐based practice implementation in acute hospital settings.

**Design:**

A focused ethnographic collective case study was conducted across two contrasting critical care units in England.

**Methods:**

Methods included non‐participant observation (56 sessions), semi‐structured interviews (36 participants) and document review. Spradley's Developmental Research Sequence guided data generation and analysis. Data were collected over an eight‐month period (February to September 2022).

**Findings:**

Five major themes were identified: sources of knowledge and acquisition strategies; institutional and hierarchical influences on knowledge use; role of experiential knowledge and clinical intuition; challenges to evidence‐based practice implementation; and strategies for integrating knowledge into practice. Organisational structures, leadership engagement, mentorship and access to updated digital resources were key enablers of evidence‐based practice. Barriers included workload pressures, inconsistent guideline dissemination and hierarchical cultures. Adaptive blending of formal evidence, clinical experience and intuition characterised effective knowledge negotiation at the bedside.

**Conclusion:**

Knowledge use in critical care nursing is a dynamic, relational process shaped by leadership, organisational culture and systemic pressures. The availability of evidence alone is insufficient; visible leadership, peer learning, protected educational time and valuing of experiential knowledge are critical to embedding evidence‐based practice into routine practice.

**Implications for Patient Care:**

Strengthening organisational systems, investing in nurse manager development, expanding simulation‐based learning and legitimising experiential knowledge are vital strategies to enhance evidence‐based critical care.

**Impact:**

This study provides actionable insights for healthcare leaders, educators and policymakers seeking to optimise evidence‐based practice adoption in high‐acuity clinical environments and improve patient outcomes.

**Reporting Method:**

The Consolidated Criteria for Reporting Qualitative Research checklist guided reporting.

**No Patient or Public Involvement:**

Patients and the public were not involved in the design, conduct, reporting or dissemination of this research.

**Summary**

- What does this paper contribute to the wider global clinical community?
  ○ Knowledge use in critical care is a dynamic, relational process shaped by organisational culture, leadership and the negotiation of evidence, experience and intuition.
  ○ Effective application of evidence depends on visible leadership, access to current resources and structured mentorship, highlighting system‐level conditions relevant across healthcare settings.
  ○ This study offers a transferable model of knowledge integration with relevance beyond the United Kingdom, providing insights for embedding evidence‐based care in high‐acuity environments internationally.

## 1 Introduction

Translating clinical expertise into everyday practice remains a critical yet often underexplored dimension of evidence‐based practice (EBP) in critical care nursing (Melnyk et al. 2014; Ominyi and Alabi 2025). While EBP is typically defined as the integration of best available evidence, clinical expertise and patient preferences (Melnyk et al. 2014; Ominyi and Alabi 2025; Møller et al. 2022), this study focuses on how expertise shaped by experience, intuition and judgement is negotiated and applied in high‐pressure environments such as intensive care units (ICUs), high dependency units (HDUs) and cardiac care units (CCUs) (Melnyk et al. 2014; Ominyi and Alabi 2025; Møller et al. 2022; Aitken et al. 2009). These settings demand rapid, context‐sensitive decision‐making, where clinical expertise frequently complements or substitutes formal guidance during situations of complexity, uncertainty and urgency (Møller et al. 2022; Aitken et al. 2009; Whitehead et al. 2020).

While EBP frameworks provide a strong foundation, knowledge use in practice is mediated by organisational culture, leadership, interpersonal dynamics and resource availability (Tucker et al. 2019). Nurses often draw on a blend of research evidence, experiential learning, intuitive judgement and peer discussion to navigate the realities of clinical care (Melnyk et al. 2014; Ominyi and Alabi 2025). Despite global initiatives to embed EBP in healthcare systems, few studies have examined how clinical expertise, an essential component of EBP, is enacted within the unpredictable and evolving conditions of critical care practice (Møller et al. 2022; Whitehead et al. 2020; Kitson et al. 2008).

## 2 Background

Critical care refers to the specialised, immediate care provided to patients with life‐threatening injuries or illnesses requiring continuous monitoring, advanced interventions and complex clinical decision‐making (Møller et al. 2022; Urden et al. 2022). This care is traditionally delivered in hospital‐based settings such as ICUs, HDUs, CCUs, Emergency Departments (EDs) and Post‐Anaesthetic Care Units (PACUs), but also extends to prehospital environments, including emergency retrieval services and military field hospitals where nurses deliver advanced life support under austere and rapidly changing conditions (Aitken et al. 2009; Whitehead et al. 2020; Tucker et al. 2019; Kitson et al. 2008; Urden et al. 2022; Benner et al. 2022; Mukuve et al. 2023).

Critical care nursing is recognised as a distinct specialisation within nursing practice and requires post‐registration education and credentialing beyond initial qualification (World Federation of Critical Care Nurses 2019). Critical care nurses across many healthcare systems complete post‐qualifying, competency‐based training to deliver safe, evidence‐based interventions for patients with critical illness (World Federation of Critical Care Nurses 2019; Ominyi et al. 2025). These nurses are expected to demonstrate expertise in physiological assessment, life support management, technological proficiency and interdisciplinary collaboration under conditions of high clinical uncertainty (World Federation of Critical Care Nurses 2019; Jackson et al. 2021).

While critical care delivered beyond hospital settings, such as in retrieval nursing or combat zones, is an important global practice (Abbott et al. 2006), this study focuses on hospital‐based critical care settings to examine the organisational and cultural dynamics shaping knowledge use. Delivering evidence‐based care in these environments depends not only on access to current clinical guidelines but also on supportive structures, engaged leadership and the capacity to integrate formal evidence with clinical experience (Whitehead et al. 2020; Abbott et al. 2006). Systemic barriers such as variability in access to education, staff shortages, high patient acuity and time constraints often limit opportunities for critical engagement with research evidence during practice (Tucker et al. 2019; Jackson et al. 2021).

Models of EBP promote structured pathways for knowledge application (Whitehead et al. 2020; Tucker et al. 2019; Kitson et al. 2008); however, the realities of clinical practice frequently demand adaptive, negotiated approaches. Nurses draw on evidence, clinical judgement, intuition and peer consultation to respond to evolving patient needs (Ominyi and Alabi 2025; Carrier 2020). Organisational factors, including leadership visibility, mentorship and team hierarchies, further mediate how evidence is interpreted and applied at the bedside (Abbott et al. 2006).

The COVID‐19 pandemic exposed and intensified these challenges, revealing vulnerabilities in knowledge infrastructure and highlighting the limitations of rigid, protocol‐driven models of EBP in acute settings (Melnyk et al. 2014; Tucker et al. 2019). These limitations have drawn attention to the importance of context‐sensitive, adaptive strategies for using knowledge in practice. Despite this growing recognition, much of the literature continues to prioritise implementation outcomes over the lived, relational aspects of knowledge use in critical care (Kitson et al. 2008).

Existing literature continues to prioritise implementation metrics over understanding the lived and relational experiences of knowledge use in critical care nursing (Whitehead et al. 2020; Tucker et al. 2019; Kitson et al. 2008; Urden et al. 2022; Benner et al. 2022). As a result, a critical gap remains in how clinical expertise, the second pillar of EBP, is translated into dynamic and context‐responsive action within high‐pressure environments. This study addresses that gap by examining how hospital‐based critical care nurses access, negotiate, and apply knowledge in their everyday practice, with particular attention to the organisational, cultural and leadership factors that shape the enactment of EBP in complex and high‐acuity settings.

## 3 The Study

### 3.1 Aims

This study aimed to explore how critical care nurses in high‐acuity hospital settings access, negotiate and apply different forms of knowledge in real‐time clinical decision‐making. Specifically, the study sought to:

- Examine how organisational, cultural and leadership contexts shape nurses' engagement with EBP at the bedside.
- Identify the sources of knowledge critical care nurses draw upon in dynamic care environments.
- Explore how educational preparation, professional development and workplace infrastructures influence the adaptation and integration of evidence‐based knowledge in critical care nursing practice.

## 4 Methods

### 4.1 Research Design

We adopted a focused ethnographic design (Knoblauch 2005) because it was well suited to exploring complex clinical practices through intensive yet time‐efficient fieldwork in modern healthcare settings (Wall 2015; Cruz and Higginbottom 2013). The study was grounded in a constructivist epistemology, recognising knowledge as co‐constructed through social interaction, experience and shared cultural meanings (Lincoln and Guba 1985; Charmaz 2014). An interpretivist theoretical perspective shaped our understanding of how nurses interpret their professional environments, supported by axiological reflexivity in which we critically considered how our clinical, academic and positional identities influenced our field engagement (Finlay 2002; Berger 2015). We assumed that reality is socially and contextually constructed, reflecting multiple truths shaped through clinical practice, organisational culture and interpersonal interaction (Finlay 2002).

The ethnographic orientation drew on traditions in social science ethnography (Silverman 2020) and selected elements from applied anthropology (Hammersley and Atkinson 2019), allowing us to use frameworks most appropriate for studying knowledge use in acute care nursing. We did not aim to follow a single ethnographic canon but rather prioritised relevance to healthcare contexts. Spradley's Developmental Research Sequence (DRS) provided the analytic structure, guiding a staged process of descriptive, focused and selective observation, aligned with our evolving understanding of knowledge use (Spradley 1980). Close attention was given to positionality and power dynamics throughout fieldwork, with the team critically examining how professional backgrounds might influence participant interactions.

While the study included collective case elements to capture variation across two contrasting hospital sites (Stake 1995; Yin 2018), its methodological identity remained firmly rooted in focused ethnography. The collective case design enabled cross‐site comparison while keeping cultural patterns of knowledge use within acute nursing practice as the central focus. Reporting followed the Consolidated Criteria for Reporting Qualitative Research (COREQ) checklist (Data S1) (Tong et al. 2007). The following section outlines how reflexivity was enacted through the research team's positioning and engagement in the field.

### 4.2 The Research Team and Reflexivity

Familiarity with the clinical setting and critical reflexivity were central to the focused ethnographic approach, enabling active engagement while supporting analytic distance (Knoblauch 2005; Higginbottom et al. 2013). All but one member of the research team was a registered nurse with academic and research roles and prior clinical experience in ICU, HDU or CCU environments. None of the researchers were practising at the study sites, which supported an open and non‐evaluative stance (Roper and Shapira 2000).

We adopted a passive learner position during non‐participant observations, minimising disruption to care delivery. Clinical guideline adherence was assessed through naturalistic observation and later triangulated with interviews and documents. At no stage did we intervene in patient care, maintaining the integrity of the focused ethnographic approach. Potential power imbalances were actively considered, with researchers positioning themselves as learners rather than evaluators (Berger 2015). Professional affiliations were disclosed transparently, and questioning during interviews remained non‐directive and open, avoiding hierarchical tone or influence.

Reflexive strategies were applied throughout the study, including continuous journaling, peer debriefing and collaborative data interpretation. These processes were essential in surfacing assumptions, monitoring researcher–participant dynamics, and ensuring that interpretations remained closely grounded in participants' lived experiences (Malterud 2001).

### 4.3 The Study Setting

This study was conducted within selected critical care units, specifically the ICUs, HDUs and CCUs of two acute care hospitals in the Midlands, England. Rather than examining entire hospital systems, the study focused on these high‐pressure units where rapid decision‐making, interdisciplinary collaboration and EBP demands are most pronounced (Møller et al. 2022; Aitken et al. 2009; Ominyi and Ezeruigbo 2019; Ominyi et al. 2025).

Hospitals were purposively selected following preliminary discussions with regional nursing leaders and education managers to capture variation in organisational structures influencing knowledge use. Specifically, sites were chosen to reflect contrasting leadership models, mentorship approaches and knowledge‐sharing cultures, providing a meaningful basis for comparative analysis. Site A was characterised by structured leadership development, formalised mentorship programmes, frequent simulation‐based learning opportunities and interdisciplinary decision‐making frameworks, while Site B operated with hierarchical leadership, informal mentorship, limited simulation opportunities and largely tacit knowledge‐sharing practices.

Focusing exclusively on ICU, HDU and CCU settings enabled the exploration of environments where critical, time‐sensitive decision‐making is integral to everyday practice, aligning with focused ethnographic principles that emphasise the study of specific cultural groups in context (Knoblauch 2005; Wall 2015). Critical care units were selected because they represent settings where the negotiation between formal evidence, clinical expertise and organisational culture is most visible and consequential for patient outcomes (Wall 2015). Access was negotiated with senior leadership teams, with site selection further guided by the feasibility of immersive engagement, institutional willingness to support prolonged fieldwork and variation in organisational commitment to EBP implementation (Wall 2015; Cruz and Higginbottom 2013). The comparative site and unit selection allowed for an in‐depth understanding of how different organisational cultures, leadership dynamics and educational infrastructures shaped critical care nurses' engagement with knowledge. A comparative overview of key organisational and cultural features is presented in Table 1.

**TABLE 1:** Organisational and cultural characteristics of the study settings.

Domain	Site A	Site B
Unit bed capacity	ICU (10 beds), HDU (12 beds), CCU (12 beds)—total 34 beds	ICU (8 beds), HDU (10 beds), CCU (10 beds)—total 28 beds
Leadership structure	Shared governance model; interdisciplinary leadership engagement.	Hierarchical leadership; top‐down decision‐making.
Knowledge dissemination	Structured, regular updates; formal training sessions.	Ad hoc, informal knowledge sharing; reliance on peer communication.
Mentorship approach	Formal preceptorship programmes; structured peer support.	Informal mentorship; organic peer learning.
Use of guidelines and protocols	Frequent access to updated guidelines; formal review processes.	Reliance on outdated printed guidelines; inconsistent updates.
Simulation and training opportunities	Regular simulation sessions and structured learning opportunities.	Limited, ad‐hoc simulation sessions; fewer structured practical learning opportunities.
Role of experiential knowledge	Experiential knowledge integrated with EBP; encouraged alongside clinical intuition.	Heavy reliance on experiential knowledge due to limited formal training opportunities.
Team culture and decision‐making	Collaborative, open team discussions encouraged.	Physician‐dominated discussions; limited nurse input.
Barriers to EBP implementation	Time pressures acknowledged but partially mitigated by leadership support.	Time pressures compounded by inconsistent knowledge structures.
Ethical and institutional compliance	Regular updates and tracking for guideline compliance and professional development.	Limited formal mechanisms for tracking guideline adherence.

### 4.4 Participants (Recruitment, Sampling and Sample Size)

A purposive sampling was employed to recruit critical care nurses working specifically within the units outlined in the section earlier (Wall 2015; Cruz and Higginbottom 2013). Sampling was designed to ensure the inclusion of staff directly involved in high‐acuity clinical practice, where EBP integration and knowledge utilisation are especially critical (Møller et al. 2020; Carrier 2020). A total of 36 participants were recruited: 26 staff nurses (SNs), six nurse managers (NMs), and four advanced clinical practitioners (ACPs). In this study, a ‘staff nurse’ referred to a registered nurse providing direct bedside care in critical care settings. ‘Nurse managers’ held clinical leadership responsibilities, while ‘ACPs’ carried advanced clinical decision‐making authority, often at a senior practice level. This grouping ensured that multiple perspectives on knowledge acquisition, dissemination and application were captured across different levels of professional responsibility. The sampling strategy was guided by the following inclusion and exclusion criteria (Table 2).

**TABLE 2:** Inclusion and exclusion criteria.

Category	Description
Inclusion criteria	Registered SNs, NMs or ACPs working in ICU, HDU or CCU settings at the selected hospitals. Minimum of two years' experience in critical care nursing to ensure familiarity with knowledge use in dynamic, high‐pressure environments. Willingness to provide informed consent for participation in observations and interviews. Ability to participate in multiple engagements over the study period.
Exclusion criteria	Temporary agency staff without sustained integration into the clinical team. Withdrawal during the study period due to work pressures or personal/health reasons. Inability to provide informed consent.

Participants' years of experience ranged from 5 to 30 years. Their highest educational qualifications ranged from Bachelor of Science (BSc) to Doctor of Philosophy (PhD) degrees. Given that formal EBP education has only been systematically embedded in nursing curricula over the past two decades, this variability was important for understanding different educational exposures to EBP.

While not a primary sampling criterion, workforce factors such as nurse‐to‐patient ratios were noted during fieldwork as influencing participants' engagement with EBP. Both hospitals reported staff shortages and increased patient loads, factors that shaped nurses' opportunities for formal knowledge acquisition and guideline implementation. These organisational conditions are discussed in the introduction and later reflected in the findings section as important contextual influences (Whitehead et al. 2020; Jackson et al. 2021). Participant demographic characteristics, including professional role, years of experience and highest qualification, are presented in Tables 3 and 4.

**TABLE 3:** Participant demographics (site A).

Participant ID	Role	Years of experience
A‐SN1	SN	5 years
A‐SN2	SN	12 years
A‐SN3	SN	18 years
A‐SN4	SN	14 years
A‐SN5	SN	19 years
A‐SN6	SN	22 years
A‐SN7	SN	7 years
A‐NM1	NM	23 years
A‐NM2	NM	28 years
A‐NM3	NM	17 years
A‐ANP1	ACP	16 years
A‐ANP2	ACP	21 years
A‐ANP3	ACP	11 years
A‐ANP4	ACP	6 years
A‐ANP5	ACP	26 years
A‐ANP6	ACP	19 years
A‐ANP7	ACP	13 years

Abbreviations: ACP, advanced clinical practitioner; NM, nurse manager; SN, staff nurse.

**TABLE 4:** Participant demographics (site B).

Participant ID	Role	Years of experience
B‐SN1	SN	6 years
B‐SN2	SN	11 years
B‐SN3	SN	17 years
B‐SN4	SN	15 years
B‐SN5	SN	18 years
B‐SN6	SN	20 years
B‐SN7	SN	8 years
B‐SN8	SN	13 years
B‐SN9	SN	16 years
B‐NM1	NM	22 years
B‐NM2	NM	29 years
B‐NM3	NM	18 years
B‐ANP1	ACP	14 years
B‐ANP2	ACP	24 years
B‐ANP3	ACP	12 years
B‐ANP4	ACP	7 years
B‐ANP5	ACP	27 years
B‐ANP6	ACP	20 years
B‐ANP7	ACP	14 years

Abbreviations: ACP, advanced clinical practitioner; NM, nurse manager; SN, staff nurse.

### 4.5 Data Collection

Data collection was conducted over an eight‐month period, between February and September 2022, following a two‐month pre‐fieldwork access and negotiation phase (November 2021–January 2022). This preparatory period included hospital approvals, field entry planning and rapport‐building activities (Knoblauch 2005; Wall 2015). The extended duration allowed for the observation of seasonal variations in hospital workflows and supported prolonged engagement, thereby enhancing data richness and trustworthiness (Hammersley and Atkinson 2019). Throughout the study, COVID‐19 infection prevention measures were strictly adhered to, including the use of Personal Protective Equipment (PPE) and the minimisation of non‐essential contact, in compliance with hospital and national guidelines (Tong et al. 2007).

A triangulated approach was used, integrating three primary methods: non‐participant observation, semi‐structured ethnographic interviews and review of documentation (Patton 2015; Bowen 2009). This combination of methods, alongside contemporaneous field notes and reflective memos, enhanced the credibility and depth of the study (Nowell et al. 2017; Braun and Clarke 2022). Each method is outlined below.

#### 4.5.1 Participant Observation

A non‐participant, overt observer role was adopted (Hammersley and Atkinson 2019; Spradley 1980), with researchers openly disclosing their observer status through ‘Observer’ badges and neutral clinical attire, ensuring both transparency and minimal disruption to clinical practice. Immersion in clinical activities was facilitated through continuous presence across day, evening and night shifts, capturing a broad range of clinical dynamics. The first author (a registered nurse academic, but naïve to the specific sites) conducted all observations, observing approximately 16 SNs, 6 NMs and 4 ACPs across the two sites.

A total of 56 observation sessions were conducted, each lasting between 3 and 5 h, covering different times of the day (morning, afternoon and night shifts) to capture the influence of temporal factors on knowledge use. Observations were distributed equally across ICU, HDU and CCU settings at both hospitals. Observations were structured following SDR research Sequence (Spradley 1980), progressing from descriptive observation (familiarisation with environment and routines) to focused observation (identifying knowledge interactions) and finally to selective observation (concentrated on specific patterns such as leadership influence, peer mentorship or evidence adaptation). To minimise the Hawthorne effect and reduce power imbalance, we consistently reaffirmed voluntary participation, maintained a ‘learner’ stance (Berger 2015) and sought permission from staff present at the beginning of each session. Impact on third parties was addressed ethically, with verbal consent reaffirmed and individuals free to opt out of observed interactions.

Field notes were recorded (Data S2), contemporaneously, documenting clinical decisions, knowledge‐sharing interactions and contextual factors influencing knowledge use (Bernard 1994). These were complemented by reflective memos, critically examining positionality, emerging biases and emotional responses (Finlay 2002). Observational judgements about adherence to clinical guidelines were not checklist‐driven but inductively inferred and later triangulated with interview data and document analysis (Spradley 1980). Exit from the field was phased, involving final feedback meetings with unit managers and thank‐you communications to participating units, ensuring ethical closure and reciprocity (Finlay 2002).

#### 4.5.2 Ethnographic Interviews

Interviewing was conducted after preliminary field immersion to allow for alignment between observed phenomena and participants' accounts. Following the observational phase, 36 semi‐structured interviews were conducted with SNs, NMs and ACPs across the two hospital sites. Interviews were strategically scheduled during and after the observation period to allow field insights to refine and enrich the interview guide (Charmaz 2014; Rubin and Rubin 2012). Both the first and third authors conducted the interviews collaboratively. Interviews were conducted in quiet, private locations within the hospitals, respecting participants' preferences for confidentiality and minimising workplace fatigue. Each interview lasted between 60 and 120 min, depending on participant availability and conversational flow.

The semi‐structured interview guide was developed based on Spradley's DRS (Spradley 1980), progressing from descriptive to structural and contrast questions. Importantly, preliminary field observations informed the refinement of interview prompts, enabling direct exploration of behaviours and patterns noted during clinical observations (e.g., informal knowledge sharing, adaptation of protocols). Interviews were scheduled following initial field engagement, allowing real‐time insights to be investigated further with participants. Selected examples from the interview guide are presented in Table 5 (Spradley 1980; Rubin and Rubin 2012). See Data S3 for further details.

**TABLE 5:** Selected sample of interview questions.

Descriptive question	Structural question	Contrast question
During your shift, what types of information do you typically use when making clinical decisions?	How do you usually incorporate new knowledge or guideline updates into your practice?	When you have to act quickly, how does following formal guidelines differ from using your own clinical experience or intuition?
Can you describe how you usually share information or updates with your colleagues during a shift?	What formal or informal routines exist in your unit to support knowledge sharing among staff?	How would you compare what happens during scheduled team meetings to the informal discussions that happen during handovers or breaks?
During observations, I noticed that updates to protocols were sometimes discussed briefly at shift handovers. Can you tell me more about how these updates are usually communicated and acted upon?	In your experience, how do staff respond when a new guideline contradicts established practices based on prior experience?	How does the unit culture influence whether people rely more on written protocols or their personal and team experiences?
During fieldwork, I observed instances where nurses adapted practice based on intuition rather than strict protocol adherence. Can you share a time when you had to do this, and what influenced your decision?	How do team dynamics, leadership styles or staffing pressures affect how evidence is used during real‐time clinical care?	How would you compare decision‐making processes during quieter shifts versus high‐acuity or short‐staffed periods?

#### 4.5.3 Review of Documentation

Review of documentation was undertaken as a core data collection method alongside participant observation and interviews, enabling a comprehensive understanding of the organisational context shaping knowledge utilisation. This process focused exclusively on hospital‐generated materials, including clinical guidelines, sepsis protocols, airway management checklists, training manuals, policy updates, mentorship frameworks, governance records and workforce audits.

Document analysis was conducted concurrently with observations and interviews to support methodological triangulation and strengthen the trustworthiness of the study. For example, the availability and currency of protocols observed during fieldwork were cross‐referenced against documented guideline revision logs, and participant accounts of guideline dissemination were compared to formal governance dissemination policies. Only institutional documents were included in this review; researcher‐generated materials, such as field notes and reflective memos, were deliberately excluded to ensure the analysis reflected authentic organisational discourses rather than researcher interpretations alone (Whitehead et al. 2020). The document review provided essential contextual data, allowing comparison between formal organisational intentions and actual clinical practices observed in the field.

### 4.6 Ethical Considerations

This study was conducted in accordance with the Declaration of Helsinki and the UK Policy Framework for Health and Social Care Research (World Medical Association and WMA 2013; Health Research Authority (HRA) 2017). Ethical approval was granted by the University Research Ethics Committee (Reference ID: #00184), with site‐specific NHS governance approvals obtained prior to fieldwork.

COVID‐19 infection prevention measures were rigorously followed, including PPE use, minimisation of face‐to‐face interactions and contingency planning for unit outbreaks. The researcher did not assume any clinical nursing role during fieldwork.

Confidentiality was safeguarded by anonymising all participant data during transcription and removing educational qualifications from demographic details where disclosure risked identifiability. Written informed consent was obtained prior to participation, with verbal assent reaffirmed at the beginning of each observation or interview session by the researcher. Non‐nursing individuals inadvertently observed were informed and given the opportunity to opt out verbally; their data were excluded unless explicit consent was obtained (Murphy and Dingwall 2007). Procedures for observing critical incidents were pre‐specified. The researcher adopted a passive, non‐interventionist stance and did not question or engage staff during emergencies, consistent with ethical guidance for high‐stakes observational research (Hammersley and Atkinson 2019).

The lead researcher maintained detailed reflexive memos, including reflections following critical events, and accessed academic supervision for debriefing support. No judgements regarding the evidence base of observed practices were made in real time; interpretations regarding EBP adherence occurred post hoc during data triangulation (Finlay 2002). Professional background disclosures were carefully scripted to emphasise the researcher's learner stance and non‐evaluative position. Participation was explicitly voluntary, and participants were offered multiple opportunities to withdraw. Any existing professional connections were declared transparently to mitigate coercion (Berger 2015).

### 4.7 Data Analysis

**FIGURE 1:**
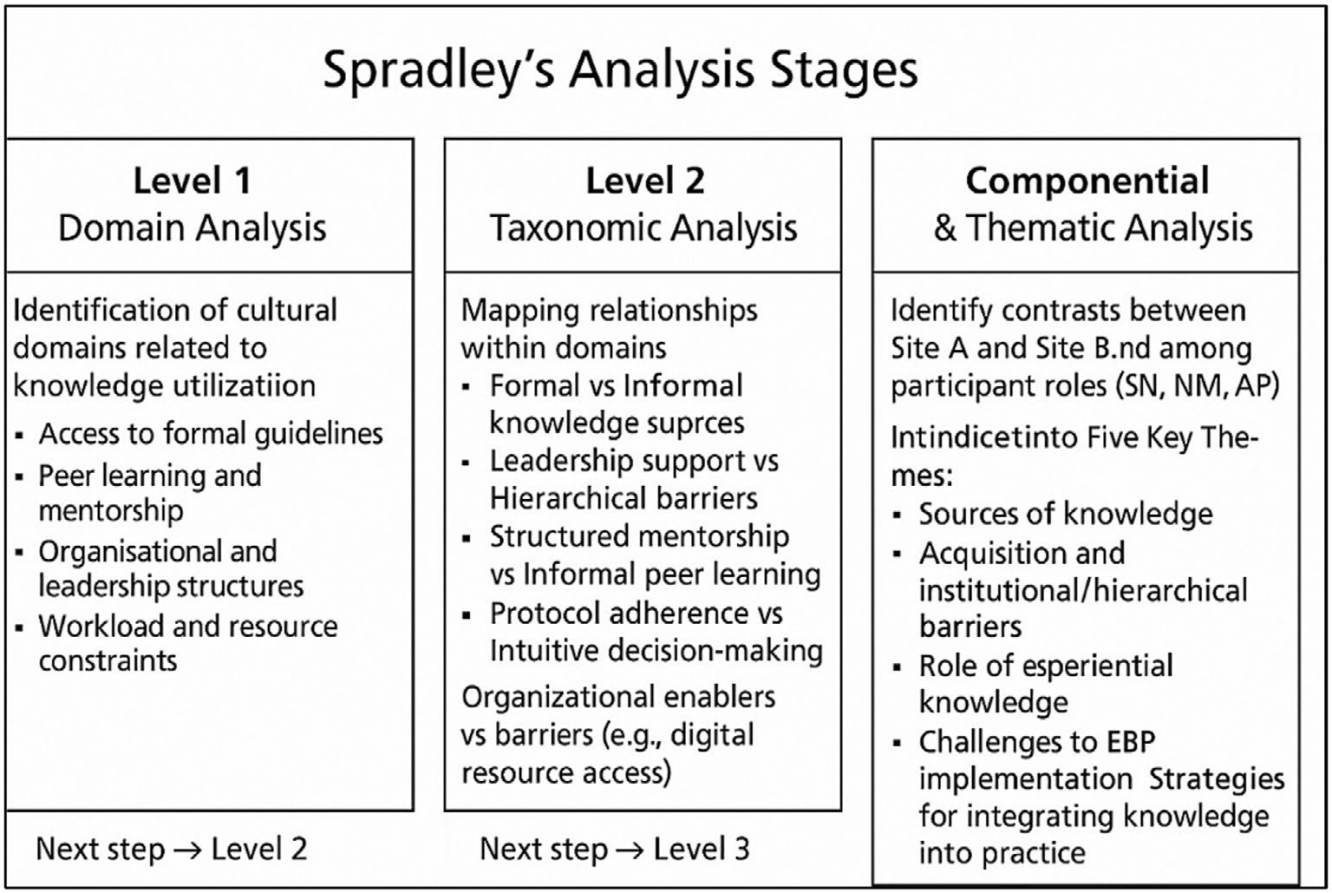
Spradley's analysis stages.

Data analysis was conducted concurrently with data collection, following an iterative and reflexive approach consistent with focused ethnography (Knoblauch 2005; Wall 2015). Early reflections informed subsequent field engagement, with emergent findings shaping ongoing data collection. The first author, a registered nurse academic experienced in EBP, ethnography, and critical care nursing, served as the primary analyst. Spradley's DRS guided the process (Spradley 1980), progressing through domain, taxonomic and componential analysis stages. Initially, broad cultural domains were identified (access to guidelines, peer mentorship, leadership structures, experiential learning and resource constraints), with NVivo 12 software supporting the organisation of cover and included terms. A sample domain structure is shown in Figure 1. Taxonomic analysis mapped internal relationships, revealing contrasts such as formal versus informal knowledge sources and structured versus informal mentorship (Hammersley and Atkinson 2019). The componential analysis further examined differences between study sites and participant roles (SNs, NMs, ACPs), particularly around guideline access, leadership practices and the operationalisation of experiential knowledge (Charmaz 2014).

Thematic synthesis then refined findings into five major cultural themes. Coding reliability was strengthened through independent cross‐coding by the third author (an experienced qualitative methodologist), with 25% of transcripts and field notes double‐coded and discrepancies resolved through team debriefings (Nowell et al. 2017; Barbour 2001). Further details about the coding frame are provided in the Data S4. Inductive open coding was initially applied line‐by‐line across interviews, observations and documents (Braun and Clarke 2022). Codes were then grouped into higher‐order categories aligned with emerging domains. Member checking at the theme development stage involved participant review of anonymised summaries to verify interpretive resonance (Birt et al. 2016).

Analysis of documentation used directed content analysis (Hsieh and Shannon 2005), triangulating observed and reported practices with organisational documents (Bowen 2009). Educational level was considered analytically but interpreted as contextual rather than causal in variations in knowledge use across the professional hierarchy. Figure 1 illustrates the staged progression from domain identification to thematic synthesis following Spradley's analytic model (Spradley 1980).

### 4.8 Rigour and Reflexivity

Trustworthiness was ensured through triangulation, a detailed audit trail (Data S5), and critical reflexivity throughout the study. Methodological and data source triangulation integrated observations, interviews and document analysis, enabling cross‐validation and identification of convergent and divergent patterns (Patton 2015; Nowell et al. 2017). Dependability and confirmability were supported by thorough documentation of analytic decisions, coding frameworks and theme development matrices. An audit trail detailing these processes is provided in Supporting Information (Lincoln and Guba 1985; Nowell et al. 2017).

Member checking (Data S6) strengthened credibility by inviting participants to review preliminary theme summaries, refining authenticity (Birt et al. 2016). Intercoder agreement was enhanced by independent cross‐coding of a purposive sample of transcripts and field notes, with discrepancies resolved through dialogue, consistent with qualitative best practice (Barbour 2001).

Reflexivity was systematically integrated, recognising the researcher's interpretive role within a constructivist epistemology (Lincoln and Guba 1985; Charmaz 2014). The lead researcher maintained a reflexive journal throughout fieldwork and analysis, documenting positionality, evolving interpretations and emotional responses (Finlay 2002; Berger 2015). Regular peer debriefings further interrogated assumptions, challenged potential biases and reinforced analytical rigour.

## 5 Findings

Findings from observations, interviews, document analysis and field journals are presented, supported by verbatim quotes and field observations. Table 6 summarises the key themes and subthemes, highlighting the diverse sources, organisational dynamics and contextual challenges shaping knowledge utilisation.

**TABLE 6:** A summary of key themes/subthemes and raw quotes.

Theme	Subtheme	Sample raw quotes
Sources of knowledge and knowledge acquisition	Access to formal guidelines and protocols	‘We have monthly clinical governance meetings where we discuss the latest guidelines’ (A‐NM2)
		Observed team briefing: laminated updated sepsis protocols distributed and discussed (Observation 22, Site A, ICU)
		Policy folders are visibly available and updated every six months, as per documentation review (Document Analysis – Site A Governance Policy)
		Protocols felt visibly embedded in daily routines at Site A but appeared absent in comparable shifts at Site B (Reflective Journal)
	Role of peer learning and mentorship	‘New nurses shadow experienced staff for a few weeks, which helps them understand how we apply knowledge in practice’ (A‐SN3)
		Observation: senior nurse coaching a new nurse on ventilator management using ‘what works best’ rather than a manual (Observation 28, Site B, CCU)
		Staff induction booklet outlines ‘informal peer support encouraged; no mandatory mentorship’ (Document Analysis – Site B Induction Booklet)
		Peer mentorship critical but variable across shifts; heavily influenced by staffing levels (Reflective Journal)
Institutional and hierarchical influences on knowledge use	Organisational culture and decision‐making	‘We're encouraged to speak up during rounds and ask questions if something feels off’ (A‐ACP3)
		Observed MDT round: nurses questioned senior consultants about antibiotic adjustments, with active discussion (Observation 9, Site A, ICU)
		Meeting minutes show open interdisciplinary contributions were agenda items at Site A governance meetings (Document Analysis)
		Nurses in Site A visibly more comfortable suggesting changes compared to Site B (Reflective Journal)
	Leadership support for knowledge use	‘Our nurse manager updates us regularly on policy changes and what it means for patient care’ (A‐NM1)
		Observation of nurse manager‐led ‘policy update huddle’ before a shift start (Observation 14, Site A, HDU)
		Site B audit: ‘No formal mechanism for ensuring guideline updates are disseminated’ (Document Analysis – Audit Report)
		Leadership pivotal in normalising guideline discussions during clinical handovers at Site A (Reflective Journal)
The role of experiential knowledge and clinical intuition	The use of intuition in clinical decision‐making	‘Sometimes, you just know when something isn't right, even before the vitals change’ (A‐SN5)
		Observation: ACP initiated rapid intervention based on ‘gut feeling’ despite normal observation (Observation 19, Site A)
		No formal documentation recorded intuition; policy documents emphasise protocol adherence (Document Analysis)
		Tension noted between ‘following gut’ and sticking rigidly to numeric criteria during observations (Reflective Journal)

	The influence of past experiences on knowledge use	‘After a similar case last year, I knew exactly what to look for’ (A‐SN8)
		Observed during cardiac arrest drill: nurse referenced previous airway difficulties to anticipate alternative interventions (Observation 25, Site A)
		Training guidelines lack explicit space for reflective learning from past cases (Document Analysis)
Time constraints and workload pressures	Senior nurses often drew directly on case memory during emergencies more than protocols (Reflective Journal)
	Challenges in evidence‐based practice implementation	‘When you're short‐staffed, there's no time to look up the latest evidence; you have to act’ (A‐ACP2)
		Observation: break rooms empty during shifts; no protected time seen for evidence consultation (Observation 16, Site B)
		Staff survey showed ‘high perceived workload’ as the main barrier to EBP training attendance (Document Analysis – Site B Workforce Survey)
		During busy shifts, the idea of stopping to check evidence seemed unrealistic – action took precedence (Reflective Journal)
	Inconsistencies in guideline dissemination	‘I only found out about a major sepsis protocol change by overhearing a colleague’ (B‐ACP6)
		No posters, guideline updates or reminders observed in staff areas at Site B (Observation 31, CCU, Site B)
		Governance audit at Site B identified ‘no formal system to track staff awareness of updates’ (Document Analysis)
		Silence around updates at Site B striking compared to Site A's structured communication routes (Reflective Journal)
Strategies for integrating knowledge into practice	Peer discussions as a knowledge‐integration strategy	‘After a complex case, we sit down and talk about what worked and what didn't’ (A‐NM3)
		Observed post‐cardiac arrest debrief with multidisciplinary input (Observation 21, Site A, ICU)
		Staff debriefing guidance outlined as mandatory after critical incidents at Site A (Document Analysis)
		Debriefs a crucial, under‐appreciated space where guidelines meet lived clinical reality (Reflective Journal)
	Simulation‐based learning and digital resources	‘We have monthly simulation drills – it helps bridge theory and reality’ (A‐ACP1)
		Observed high‐fidelity simulation session on sepsis management (Observation 17, HDU, Site A)
		Simulation attendance logs mandatory for Site A ICU/HDU nurses; optional for Site B (Document Analysis)
		Sim sessions visibly boosted nurses' confidence in adapting new guidelines on the ward (Reflective Journal)

**FIGURE 2:**
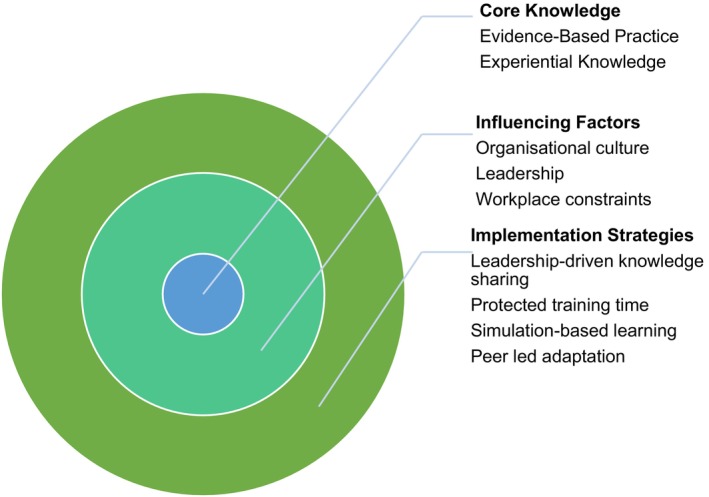
Multi‐layered framework of knowledge use in critical care nursing.

Building on these insights, Figure 2 conceptualises knowledge use as a dynamic, layered process.

The framework is organised into three concentric layers, each carrying distinct significance. The core layer represents *bedside knowledge negotiation*, the active, adaptive integration of formal evidence, experiential learning, clinical intuition and patient context during real‐time clinical decision‐making. The middle layer captures *organisational and hierarchical influences*, such as leadership support, team dynamics, mentorship structures and the dissemination of clinical guidelines. These factors directly mediate and shape how knowledge negotiation unfolds. The outermost layer represents *broader systemic and contextual influences*, including workforce pressures, education and training backgrounds and wider health system structures. While these factors are more distal, they create the enabling or constraining environment in which organisational practices and individual knowledge negotiation occur.

The layered placement is deliberate: proximity to the core indicates the *directness and immediacy* of influence on frontline clinical decision‐making. Organisational influences operate one step removed, while systemic factors exert broader but less immediate pressures. The framework portrays knowledge utilisation not as a linear application of evidence but as a negotiated, multi‐level process shaped by individual, organisational and systemic forces.

### 5.1 Sources of Knowledge and Knowledge Acquisition

#### 5.1.1 Access to Formal Guidelines and Protocols

Access to formal guidelines was routinely supported by structured governance mechanisms in Site A. Monthly clinical governance meetings enabled staff to discuss and integrate updated guidelines into everyday practice. As one nurse manager explained,

We have monthly clinical governance meetings where we discuss the latest guidelines…if there's an update, we are briefed immediately, and this helps ensure we apply evidence correctly (A‐NM2).

Observations at Site A confirmed this culture:

In one team briefing, laminated copies of updated sepsis protocols were distributed and discussed openly. The nurses were encouraged to ask questions and clarify how the changes would affect their practice (Observation 22, Site A, ICU).

Policy folders were prominently available on wards and were reviewed every six months, as corroborated through document analysis (Site A Governance Policy).

In contrast, Site B demonstrated considerable inconsistency in the dissemination and uptake of formal knowledge sources. Nurses often described informal peer interactions as their primary method of gaining new knowledge. One registered nurse commented:

If I'm unsure about something, I ask a senior nurse or the doctor […] they usually explain it based on their experience. We don't have a lot of formal training sessions, so most of what I learn is from watching others and picking things up during shifts, especially when things get busy (B‐RN4).

Observations at Site B reinforced this, with nurses often seen referring to aged, annotated printed protocols at nursing stations. For example, a protocol manual over three years old was observed in active use during a clinical consultation (Observation 27, Site B, HDU). The absence of visible, updated resources was also recorded in reflective field notes, noting that:

protocols felt visibly embedded in daily routines at Site A but appeared absent in comparable shifts at Site B (Field Note, FN03, Site A).

Document analysis highlighted Site A's systematic updating practices, while Site B's policies simply recommended that ‘guidelines should be updated periodically’, without specifying a timeframe, leading to variations in staff access and awareness.

This contrast in knowledge accessibility fundamentally influenced the integration of EBPs across the two sites. The more formalised and visible dissemination in Site A appeared to support a more consistent application of updated evidence into patient care compared to the ad‐hoc, colleague‐reliant approach predominant in Site B.

#### 5.1.2 Role of Peer Learning and Mentorship

Peer learning and mentorship emerged as significant influences on knowledge acquisition in both sites, albeit implemented differently. Site A operated formal preceptorship programmes where new nurses were paired with experienced mentors for a defined period. One senior nurse noted:

New nurses shadow experienced staff for a few weeks, which helps them understand how we apply knowledge in practice. It's not just about learning the steps; it's also about picking up the judgement calls and small adjustments that you can't really learn from reading guidelines alone (A‐SN3).

Observations recorded structured one‐to‐one coaching, such as during a session on ventilator management where an experienced nurse systematically explained procedures while referencing current protocols (Observation 12, Site A, HDU). In Site B, mentorship occurred more informally. Rather than structured preceptorship, peer support was encouraged but not mandated. This was evident in Site B's staff induction booklet which stated,

informal peer support encouraged; no mandatory mentorship (Document Analysis, Site B Induction Booklet).

Observations highlighted that knowledge transfer was highly reliant on interpersonal relationships and staffing levels. During an evening shift, a senior nurse guided a newly recruited nurse through a critical care case based on prior experiences, using verbal instruction rather than referencing any written protocol (Observation 28, Site B, CCU). Peer mentorship was acknowledged as critical but variable.

We learn from each other a lot. If one person attends a training, they usually pass the information along to the rest of us informally. Sometimes it's just a quick chat during a handover or when we're doing paperwork, but it makes a difference because otherwise we wouldn't always hear about new things (B‐NM3).

However, the quality and consistency of this informal system fluctuated. Reflective field notes captured the observation that:

…peer mentorship is heavily influenced by staffing levels; it flourishes during quieter shifts but diminishes during high workload periods (Field Note, FN08, Site B).

While peer learning promoted rapid, context‐sensitive knowledge exchange, it also created potential gaps in evidence‐based standardisation. Site A's structured mentorship mechanisms offered more consistent reinforcement of formal evidence, while Site B's informal model risked a greater reliance on anecdotal knowledge.

#### 5.1.3 Barriers to Knowledge Access

Despite structured mechanisms in place in Site A, and ad‐hoc mechanisms in Site B, barriers to accessing knowledge were prevalent across both hospitals. Shift timing, workload pressures and digital resource availability all impacted nurses’ ability to engage with formal evidence.

Night shift staff reported particular challenges. As one senior nurse described:

…Night shifts are tricky. We don't have time to sit and review guidelines, so we rely on what we know from past experiences […] you might hear about an update during the day, but at night, you're mostly going off what you remember and what feels right at the time (A‐SN7).

Observations supported this: during night shifts, no engagement with guidelines was seen, and task prioritisation took precedence. In Site B, access to digital resources such as online guidelines and hospital intranet updates was limited, especially during off‐peak hours. One nurse highlighted:

We don't always get updates on new guidelines, so we just use what we already know. Sometimes you hear there's been a change weeks after it's happened. It's not that we don't want to use new evidence…we just don't always know it's there (B‐SN6).

Site B's document analysis confirmed a lack of structured digital resource access onwards, further compounded by staff surveys identifying ‘technology availability’ as a recurring issue (Document Analysis, Site B Staff Survey).

Observational data indicated that even when digital resources were technically available, high workload often prevented their utilisation. One observation recorded a nurse bypassing the online portal to manage an emergency admission, relying instead on ‘*what usually works*’ (Observation 29, Site B, ICU). Hospital policy documentation revealed additional discrepancies. Site A mandated six‐monthly reviews of all clinical guidelines, with sign‐off records kept. In contrast, Site B simply recommended ‘periodic review’, leading to inconsistencies in staff awareness and application of updates. Reflective notes captured this gap:

Site A's systematic updating embedded knowledge as part of professional culture; Site B's sporadic updating left knowledge refreshment to chance… (Field Note, FN12, Site A).

These barriers suggest that while individual motivation and team culture supported knowledge utilisation to an extent, structural enablers such as protected learning time, accessible technology and regular updates were crucial determinants in the consistent application of evidence‐based practice.

### 5.2 Institutional and Hierarchical Influences on Knowledge Use

#### 5.2.1 Organisational Culture and Decision‐Making

Organisational culture shaped whether nurses felt empowered to use evidence proactively or whether they deferred to traditional authority hierarchies. In Site A, nurses described a culture where knowledge‐sharing and questioning were encouraged as part of daily practice. As one Advanced Clinical Practitioner explained:

We're encouraged to speak up during rounds, and if something doesn't sit right, we can ask questions. The consultants take our input seriously and we work as a team. It makes you feel like your knowledge and observations matter, not just following orders (A‐ACP3).

Observations during multidisciplinary team meetings at Site A confirmed this dynamic. Nurses actively participated in discussions around patient management, offering evidence‐based suggestions and raising clarifications about guideline interpretations (Observation 9, Site A, ICU). Meeting minutes reviewed at Site A supported this, listing nursing contributions as standard agenda items (Document Analysis, MDT Meeting Minutes, Site A). In contrast, Site B presented a more hierarchical structure, where nurses were expected to follow directions rather than question clinical plans. As one senior nurse put it:

We're expected to follow instructions, not question them. Even if a guideline doesn't quite fit the situation, we just go with it. You don't want to be seen as difficult, even if you think another approach might be better (B‐SN7).

During an observed MDT meeting in Site B, nurses were mostly silent, with consultants driving decision‐making and minimal interdisciplinary dialogue (Observation 12, Site B, CCU). Reflective field notes highlighted the visible discomfort among some junior nurses when controversial cases arose, suggesting that institutional culture at Site B limited critical engagement with evidence‐based decisions (Field Note, FN05, Site B).

The differences between sites illustrate how organisational culture either reinforced or undermined nurses' agency in negotiating evidence use. In Site A, open cultures promoted shared ownership of EBP. In Site B, hierarchical dynamics restricted flexibility, with knowledge seen as flowing top‐down from physicians.

#### 5.2.2 Leadership Support for Knowledge Use

Leadership, particularly the role of NMs, was critical in either promoting or hindering knowledge dissemination and EBP. In Site A, participants consistently reported that NMs actively facilitated access to updated guidelines and promoted reflective practices around evidence use:

Our nurse manager always updates us on changes to policies and makes sure we understand how to implement them. She breaks it down into what it means for us on the floor, not just the technical changes. It helps because it feels more connected to our day‐to‐day work (A‐NM1).

This approach was mirrored in observations where nurse managers led regular pre‐shift policy huddles discussing evidence updates (Observation 14, Site A, HDU). Document analysis of Site A's governance policies further revealed structured responsibilities for NMs to circulate evidence updates and maintain a record of staff familiarisation (Document Analysis, Site A Policy Update Logs). In Site B, leadership practices were more inconsistent. While some managers were proactive, others relied on informal updates, leading to knowledge gaps among frontline staff. One ACP described this experience:

…Sometimes we find out about changes to protocols after they've already been in place for weeks. It depends who you're working with. Some managers are brilliant at passing things on, others just assume you know. There's no official system to make sure everyone's in the loop (B‐ACP2).

An internal audit report at Site B corroborated this concern, noting a lack of formal mechanisms for tracking staff awareness of guideline updates (Document Analysis—Site B Audit Report). During field observations, this inconsistency manifested in variability between shifts: some teams were aware of guideline changes, while others continued to follow outdated practices.

Reflective journals highlighted that at Site A, leadership visibility and accessibility enhanced a culture where EBP was seen as integral to daily practice (Field Note, FN01, Site A). At Site B, the absence of structured leadership support was associated with greater variability in knowledge use and greater reliance on experiential or tacit learning to fill formal knowledge gaps (Field Note, FN08, Site B).

### 5.3 The Role of Experiential Knowledge and Clinical Intuition

#### 5.3.1 The Use of Intuition in Clinical Decision‐Making

Participants across both sites described clinical intuition as an essential, though often under‐recognised, element of safe and responsive care. Nurses spoke of the ability to detect subtle changes in patient conditions based on experiential awareness rather than solely relying on numerical indicators. As one nurse reflected:

Sometimes, you just know when something isn't right, even before the vitals change. It's about reading the patient, not just the numbers. You notice small things…a look in their eyes, a change in their breathing that tell you something's wrong before the machines catch up (A‐SN5).

Observation data corroborated this, with one instance in Site A where an ACP initiated early intervention after sensing that a postoperative patient appeared unusually drowsy and pale, despite normal early warning scores (Observation 19, Site A, ICU). The intervention prevented subsequent deterioration, highlighting the clinical value of intuition in complementing formal monitoring systems.

Document analysis of clinical governance policies at Site A indicated a strong emphasis on protocol adherence but made no explicit reference to the role of clinical intuition, suggesting a potential misalignment between policy expectations and practice realities (Document Analysis—Site A Clinical Governance Manual). Reflective field notes captured this tension, noting that while intuition was openly valued in conversation among staff, its use remained largely undocumented and therefore invisible within formal audit processes (Field Note, FN07, Site A).

In contrast, in Site B, the organisational environment appeared less supportive of intuition‐led practice. Nurses described how strict adherence to protocols sometimes discouraged proactive clinical action. One ACP explained:

We have protocols for everything, but patients don't always fit neatly into them. Sometimes, we have to go with what we know works from experience, but even then you have to wait for a doctor to sign off, even if it's urgent. It slows things down sometimes (B‐ACP4).

Observations at Site B indicated that even when nurses perceived early signs of deterioration, there was often a hesitation to escalate care without clear, documented clinical deterioration (Observation 24, Site B, CCU). This caution reflected a cultural emphasis on defensible, protocol‐based practice over adaptive, experience‐based decision‐making.

#### 5.3.2 The Influence of Past Experiences on Knowledge Use

Past clinical experiences shaped how nurses approached both routine and emergency scenarios, providing a vital experiential resource that supplemented formal evidence. Participants across both sites reflected on the importance of case memory when managing complex or rapidly evolving patient conditions. One staff nurse commented:

After a similar case last year, I knew exactly what to look for. It's not something you learn in a book; you remember what went wrong before, and it stays with you. That way, you can act faster the next time and maybe prevent things getting worse (A‐SN8).

During an observation at Site A, a staff nurse attending to a patient with suspected sepsis recognised atypical early signs based on a prior clinical encounter, prompting the team to escalate care promptly (Observation 25, Site A, ICU).

The nurse explained that, in a previous case, a patient had deteriorated rapidly after displaying similar subtle signs. This use of past experiential knowledge directly influenced a positive clinical outcome. The observed event involved a fully consented participant and occurred naturally during routine clinical work. The researcher's role remained strictly non‐participatory throughout, with no intervention or prompting.

Document analysis at Site A revealed that while structured simulation debriefs occurred, there were limited formalised opportunities for staff to capture and disseminate learning from lived clinical experience (Document Analysis, Site A Education Strategy). As noted in the reflective journal, this informal reliance on case memory appeared to be a highly valued but largely undocumented dimension of practice. In Site B, past experiences were also cited as influential, but organisational norms shaped how freely this knowledge could be operationalised. One nurse described:

I remember dealing with a similar case last year, but here we always have to double‐check before making changes. You can't just go with your gut, even if you're sure, you have to go through the chain of command. It can slow things down a lot (B‐SN8).

Observation data at Site B (Observation 29, Site B, HDU) recorded an instance where an experienced nurse, recalling a previous airway management difficulty, identified early signs of obstruction. However, rather than immediately initiating an alternative approach, she sought registrar approval before proceeding, in line with site protocols. This cautiousness, while aiming to ensure safe practice, potentially introduced delays. Overall, experiential knowledge served as a critical, though sometimes constrained, form of evidence across both sites. Where organisational cultures were more enabling, as in Site A, past experiences were integrated into responsive care more fluidly. Where hierarchical constraint was stronger, as in Site B, reliance on previous cases often required additional.

### 5.4 Challenges in EBP Implementation

#### 5.4.1 Time Constraints and Workload Pressures

One of the most persistent barriers to EBP identified across both sites was the impact of workload pressures. Nurses repeatedly described how high patient acuity, staffing shortages and shift demands left little time for proactive engagement with evidence‐based resources. As an ACP explained:

We're expected to follow best practices, but realistically, when you have a full ward, there's no time to sit down and look up the latest evidence. You're prioritising patient care minute by minute…evidence‐based practice is great in theory, but real life doesn't always give you the space for it (A‐ACP2).

Observation data supported these accounts. During multiple shifts at Site B, break rooms remained empty for prolonged periods, and no protected time for evidence consultation was evident (Observation 16, Site B, CCU). Nurses prioritised immediate clinical tasks, and educational opportunities were frequently sacrificed to maintain patient care demands.

Document analysis of the Site B workforce survey corroborated these observations, with 78% of respondents citing ‘high perceived workload’ as the primary barrier to EBP training attendance (Document Analysis, Site B Workforce Survey). Similarly, in Site A, while better staffing ratios were noted, heavy caseloads during peak times still constrained EBP engagement, particularly during night shifts when fewer senior staff were available.

Reflective notes highlighted that even in Site A, the ideals of EBP clashed with operational realities. During one observed night shift, a senior nurse verbally acknowledged the need to review new sepsis protocols but added:

We just have to get through the night first…there's no time for anything else (Reflective Journal, Site A).

This pragmatic prioritisation of immediate patient needs over knowledge engagement was a recurrent, understandable tension across both sites. Ultimately, while commitment to evidence‐based principles was strong, the structural realities of clinical work left little room for the luxury of regular, reflective knowledge consultation during shifts.

#### 5.4.2 Inconsistencies in Guideline Dissemination

Another major challenge observed was the inconsistent dissemination of clinical guidelines, particularly at Site B. While Site A had formal structures for rolling out and updating protocols, including mandatory staff briefings and sign‐off sheets, Site B lacked a systematic process for ensuring that all nurses received and understood new evidence updates. One ACP at Site B recalled:

I found out about a major change in sepsis management weeks after it had been implemented, just by overhearing a conversation in the break room. There wasn't any formal email, no meeting, nothing. It's worrying because you wonder what else you might have missed (B‐ACP6).

Observations confirmed the absence of visual or verbal reminders of updated guidelines in Site B's clinical areas. No posters, update emails or team briefings were observed during multiple shifts (Observation 31, Site B, CCU). This stood in stark contrast to Site A, where laminated copies of critical updates were distributed and discussed during team briefings (Observation 22, Site A, ICU).

Document analysis reinforced this discrepancy. Governance records from Site A outlined a robust system whereby all new or amended guidelines were disseminated monthly and staff acknowledgements were logged (Document Analysis—Site A Policy Dissemination Record). In contrast, a governance audit at Site B noted: ‘No formal mechanism to track staff awareness of clinical guideline updates’ (Document Analysis – Site B Governance Audit).

Reflective field notes described the cultural silence around guidelines at Site B, where knowledge of changes appeared to depend on ad‐hoc conversations rather than structured communication:

Silence around updates at Site B striking compared to Site A's visible, normalised dissemination routes (Field Note, FN16, Site A).

This inconsistency in knowledge dissemination created vulnerabilities in practice, leaving some nurses unaware of critical updates and relying on outdated or informal sources of information.

#### 5.4.3 Resistance to Change

Resistance to adopting new practices, particularly those that challenged long‐standing habits, was another significant barrier to EBP implementation. Participants across both sites noted that some colleagues, particularly more experienced nurses, were reluctant to modify their practice even when new evidence emerged. As one staff nurse commented:

Some nurses stick to what they've been doing for years, even when there's new evidence suggesting a better approach (A‐SN4).

This was particularly apparent during observed training sessions at Site B, where senior nurses openly questioned the need for new protocols. For instance, during a wound care update session, a senior nurse asked,

Why change something that's worked perfectly well for decades? (Observation 30, Site B, HDU).

Document analysis from Site A's education strategy showed explicit acknowledgement of the challenge of practice change: ‘Embedding new protocols requires structured reinforcement and active leadership engagement’ (Document Analysis – Site A Education Strategy).

Site B documents, however, made no specific reference to change management strategies within clinical education plans. Resistance to change was not always overt; at times, it manifested as quiet non‐compliance or passive resistance. Reflective journal entries noted that some staff would nod through new information during huddles but later revert to familiar routines when under pressure: ‘New guidance accepted publicly but default to old ways seen repeatedly under stress’ (Field Note, FN10, Site B).

Leadership style played a mediating role. In Site A, nurse managers more proactively modelled and reinforced the adoption of new evidence, whereas in Site B, frontline staff described leadership as less visible and less engaged in promoting practice change.

### 5.5 Strategies for Integrating Knowledge Into Practice

#### 5.5.1 Peer Discussions as a Knowledge‐Integration Strategy

Informal peer discussions emerged as a primary and highly valued mechanism for integrating knowledge into practice. Participants across all groups described how conversational knowledge‐sharing after critical incidents or during breaks created opportunities to contextualise guidelines and refine clinical judgement. As one nurse manager explained:

…after a complex case, we sit down and talk about what worked and what didn't. That's where the real learning happens. It's honest…you can admit mistakes or things you didn't know…and it's where you pick up tips you'll actually use again (A‐NM3).

This was supported by observational data from Site A, where post‐incident debriefs were consistently incorporated into clinical routines. For example:

…after a cardiac arrest event, a multidisciplinary debrief was held where both nurses and doctors reflected on the decisions made, linked them to current guidelines, and identified areas for improvement (Observation 21, Site A, ICU).

Document analysis further revealed that:

Site A's governance policy mandated debriefing sessions after all critical incidents, positioning these discussions as a formalised part of learning processes (Document Analysis, Site A Governance Policy).

By contrast, Site B had no formal debriefing requirement, and observations suggested that post‐incident reflections occurred sporadically and informally, often in staff rooms or corridors rather than in structured meetings. Peer discussions also acted as a corrective mechanism for gaps in formal knowledge dissemination. For instance, in Site B, where official updates were inconsistently shared, several nurses recounted relying on conversations with colleagues to learn about protocol changes.

…you often hear about updates over coffee or during handover. There's no proper announcement or email…it's just whoever you happen to be talking to. If you miss a shift or two, you can easily miss important updates (B‐SN7).

Reflective field notes highlighted that while Site A's structured debriefings enhanced knowledge sharing, Site B's informal discussions often depended on interpersonal dynamics and were vulnerable to omissions: *Peer learning fills the gaps at Site B, but depends heavily on who you happen to be working with* (Field Note, FN02, Site B).

#### 5.5.2 Simulation‐Based Learning and Digital Resources

Simulation‐based learning was another key strategy used to reinforce evidence‐based knowledge, particularly at Site A, where it was more systematically embedded into professional development programmes. Simulation sessions provided a safe space for nurses to practise applying guidelines to complex scenarios, improving both technical skills and clinical reasoning. An ACP from Site A reflected:

We have monthly simulation drills. It helps bridge theory and reality. You get to practise the guidelines, but also deal with the messy, unpredictable stuff that happens with real patients. It gives you confidence that you're doing the right thing under pressure (A‐ACP1).

Observations confirmed that:

…simulation sessions were well‐attended, highly interactive, and focused on high‐risk scenarios such as sepsis management, cardiac arrests, and airway emergencies… (Observation 17, Site A, HDU).

Sessions involved interdisciplinary participation, and nurses were encouraged to reflect on their actions in relation to formal guidelines during debriefs immediately afterwards. Document analysis showed that participation in simulation sessions was mandatory for ICU and HDU nurses at Site A, with attendance records maintained and linked to professional appraisal processes (Document Analysis—Site A Training Compliance Log). By contrast, in Site B, simulation training was offered on an ad‐hoc basis and was neither mandatory nor consistently available due to resource limitations. As one Site B senior nurse noted:

…we're expected to know the latest protocols, but we don't get enough practical training to apply them properly. You can read about it all you want, but unless you practise it, it's easy to freeze or second‐guess yourself when you're actually faced with a real emergency (B‐SN7).

Observational data at Site B corroborated these accounts. During an unannounced visit to a clinical area, no scheduled simulation activities were noted, and staff described simulation as a ‘*luxury’* rather than a routine educational tool (Observation 23, Site B, CCU). In addition to simulation, digital resources such as online learning platforms, clinical apps and intranet‐accessible guidelines were recognised as valuable tools for knowledge integration. However, digital access varied significantly between the sites. In Site A, nurses had ready access to tablets and workstations with up‐to‐date resources, while in Site B, technological access was more limited, often requiring nurses to leave clinical areas to access computers. Reflective journal entries indicated that ready access to digital resources in Site A created a visible culture of immediate evidence consultation:

Seeing nurses quickly pull up the latest fluid management guidance mid‐ward round was normalised at Site A, far less so at Site B (Field Note, FN11, Site A).

Collectively, the structured use of simulation‐based learning and accessible digital resources played a crucial role in bridging the gap between theory and practice, reinforcing EBP principles, and enhancing nurses' confidence in adapting guidelines to real‐world clinical contexts.

## 6 Discussion

This study reveals that while access to formal evidence was available at both sites, the ability to adapt and apply evidence at the bedside depended heavily on visible leadership, team culture and operational realities. Although both study sites offered access to formal evidence‐based materials, the accessibility, visibility and operationalisation of this knowledge varied markedly, influencing nurses' capacity to integrate evidence into practice.

Consistent with prior research (Saunders et al. 2019; Craig et al. 2021), Site A's structured systems, such as mandatory clinical governance meetings, systematic protocol updates and formal preceptorships, enabled a more consistent and visible engagement with evidence‐based knowledge. Formal guidelines were not only made accessible but actively embedded into clinical routines through leadership reinforcement and institutional culture. Nurses described, and observations confirmed, an environment where knowledge updates were normalised and expected within team interactions, a finding aligning with Ominyi and Alabi's (Ominyi and Alabi 2025) argument that organisational structures are key enablers of knowledge mobilisation in high‐pressure settings.

In contrast, Site B's informal, sporadic approach to guideline dissemination, mentorship and evidence discussion highlights the risks identified by Greenhalgh et al. (2018) that without systematic reinforcement, knowledge uptake becomes contingent on interpersonal relationships and individual initiative. Informal peer learning, although valued, emerged as vulnerable to inconsistencies, omissions and anecdotal drift, particularly under conditions of staffing pressures and time constraints. These findings expand existing literature by demonstrating how overreliance on informal knowledge pathways, while fostering collegiality, can unintentionally entrench inequities in evidence access and uptake across teams.

Crucially, this study demonstrates that the mere presence of formal evidence does not guarantee its effective application in clinical practice. In the absence of structured systems to update, reinforce and embed evidence within everyday workflows, nurses frequently reverted to experiential knowledge and intuitive decision‐making, particularly during night shifts and high‐pressure periods. This finding reflects Perry's (Perry 2017) argument that the realities of contemporary nursing are shaped by competing demands, time scarcity and a need to act swiftly in uncertain contexts. Under such conditions, the practical enactment of EBP is mediated less by access to information and more by the structural and cultural conditions that support or constrain its use. Even in Site A, where resources and governance mechanisms were more robust, knowledge mobilisation remained dependent on workload and shift intensity. Without protected time for learning and seamless access to digital resources at the point of care, even well‐intentioned systems struggled to sustain consistent evidence uptake.

Importantly, this study contributes novel insights by demonstrating that organisational cultures that visibly legitimise experiential and intuitive knowledge, rather than treating it as oppositional to formal evidence, create more adaptive, resilient knowledge ecosystems. In Site A, intuitive practices and case memory were respected and informally integrated alongside protocol use. In Site B, a more rigid adherence to formal guidelines sometimes suppressed proactive clinical intuition, delaying interventions in critical situations. This finding supports emerging arguments (Black et al. 2020; Ominyi and Agom 2019) that adaptive, flexible knowledge frameworks blending formal evidence with clinical reasoning and experience are essential for safe, responsive care in dynamic acute settings.

Findings highlight the materiality of knowledge access: the physical presence of updated, user‐friendly guidelines, simulation opportunities, digital platforms and regular peer learning spaces mattered deeply. In Site A, nurses could quickly retrieve up‐to‐date protocols during ward rounds via digital devices, normalising immediate evidence consultation. In Site B, accessing digital resources was difficult, requiring nurses to leave clinical areas, an impractical option during periods of high acuity. These findings echo concerns by Tucker et al. (Tucker et al. 2019) that technological infrastructures remain uneven across NHS contexts, exacerbating disparities in EBP engagement.

This study contributes new knowledge by showing that knowledge acquisition and use in clinical practice are relational, material and processual, shaped by culture, leadership and workflow, rather than simply transferring information from publication to practice. It highlights how variability in mentorship, leadership engagement and visible knowledge infrastructures leads to uneven EBP enactment, reinforcing the need for proactive, participatory knowledge translation strategies (Black et al. 2020). It also emphasises the importance of valuing experiential and intuitive knowledge alongside formal evidence, challenging models that overly prioritise standardisation. Overall, sustainable improvements in knowledge use require investment not only at the system level but also in fostering unit‐level cultures, mentorship, access and leadership that embed evidence into relational, evolving practice.

### 6.1 Strengths and Limitations

This study offers several strengths. By integrating cultural, organisational and leadership factors, it provides a situated understanding of EBP in critical care. The focused ethnographic case study design, using interviews, observations and document analysis (Knoblauch 2005; Wall 2015; Hammersley and Atkinson 2019; Patton 2015), enabled strong triangulation and multi‐layered insights (Nowell et al. 2017; Braun and Clarke 2022). Including two contrasting hospital sites strengthened cross‐site comparisons and enhanced the contextual depth of findings (Stake 1995; Yin 2018).

However, the study's focus on two hospitals within a single region may limit broader transferability (Wall 2015). The ethnographic sample size, while rich, constrained the diversity of perspectives, and the emphasis on nursing experiences excluded wider interprofessional views. Despite reflexivity measures, researcher influence during observations cannot be entirely ruled out. Finally, although rigorous infection control procedures were followed, it is acknowledged that pandemic‐era clinical pressures may have further exacerbated time constraints and knowledge dissemination challenges during the study period.

## 7 Conclusion and Implications for Policy and Future Research

This focused ethnographic study highlights that knowledge utilisation in critical care nursing is a dynamic, relational process shaped by formal evidence, clinical experience, leadership practices and organisational culture. Nurses adapt and negotiate between research evidence, experiential learning, clinical intuition and peer consultation to manage complex, high‐acuity clinical demands. Although formal guidelines were accessible, structural barriers, including workload pressures, staffing constraints, inconsistent guideline dissemination, and hierarchical team dynamics, limited opportunities for reflective engagement with evidence at the bedside.

Findings highlight the need for healthcare organisations to build structured, visible systems to support EBP dissemination, reinforced through active leadership, mentorship and protected learning time. Investment in nurse manager development is critical to foster EBP facilitation, promote reflective cultures and legitimise experiential knowledge alongside formal evidence. Expanding access to simulation‐based learning and ensuring accessible digital resources are essential strategies to strengthen the real‐time application of evidence in critical care settings. Education providers must further enhance clinical reasoning and EBP integration within undergraduate and postgraduate nursing curricula to prepare nurses for the complex realities of modern acute care.

Future research should examine how organisational interventions support the sustained use of EBPs in critical care over time. Comparative studies across varied healthcare contexts can offer deeper insights into how leadership, interprofessional relationships and systemic structures influence knowledge mobilisation. Investigating how patient perspectives are integrated into bedside decision‐making would further enhance understanding of relational knowledge use. Advancing this agenda is essential to strengthening resilience in critical care, improving patient outcomes and ensuring that EBP is meaningfully aligned with the realities of clinical work.

## Author Contributions

Jude Ominyi, Ukpai Eze, David Agom made substantial contributions to conception and design, or acquisition of data, or analysis and interpretation of data; Jude Ominyi, Ukpai Eze, David Agom, and Aaron Nwedu given final approval of the version to be published. Each author has participated sufficiently in the work to take public responsibility for appropriate portions of the content; Jude Ominyi, Ukpai Eze, David Agom, Adewale Alabi, and Aaron Nwedu involved in drafting the manuscript or revising it critically for important intellectual content and agreed to be accountable for all aspects of the work in ensuring that questions related to the accuracy or integrity of any part of the work are appropriately investigated and resolved.

## Disclosure

Data confirmation: Confirm that any data utilised in the submitted manuscript have been lawfully acquired in accordance with the Nagoya Protocol on Access to Genetic Resources and the Fair and Equitable Sharing of Benefits Arising from Their Utilisation to the Convention on Biological Diversity. State that the relevant fieldwork permission was obtained and list the permit numbers.

## Ethics Statement

The study was conducted in accordance with the principles outlined in the Declaration of Helsinki. Ethical approval was obtained from the University Research Ethics Committee (Reference ID: #00184) and through the appropriate research governance system (Reference: #00567). Additional approval was secured from the Research and Development Units of the participating hospital sites, ensuring compliance with institutional governance protocols.

## Consent

Informed consent to participate was obtained from all participants prior to their enrolment in this study. Participant information sheets and consent forms were provided in advance via email. These documents were then reviewed and verbally confirmed at the start of each observation episode and interview recording.

## Conflicts of Interest

The authors declare no conflicts of interest.

## Supporting information


Data S1.

## Data Availability

The data that support the findings of this study are available from the corresponding author upon reasonable request.
