# Supplementary material for: Implementing Evidence‐Based Practice in Critical Care Nursing: An Ethnographic Case Study of Knowledge Use
Source: J Adv Nurs. 2025 Jul 4;82(3):2407–26. doi: 10.1111/jan.70054 (PMC12907619; doi:10.1111/jan.70054)
Supplement: Supplementary file 1 — Data S1. [file JAN-82-2407-s001.docx]

**Supplementary File 1: COREQ checklist**

Consolidated criteria for reporting qualitative studies (COREQ): 32-item checklist
Developed from:
Tong A, Sainsbury P, Craig J. (2007) Consolidated criteria for reporting qualitative research (COREQ): a 32-item checklist for interviews and focus groups. *International Journal for Quality in Health Care*, 19(6): 349–357.

| **Item No** | **Guide Questions/Description** | **Reported on Page #** |
| --- | --- | --- |
| **Domain 1: Research team and reflexivity** |  |  |
| **Personal Characteristics** |  |  |
| 1 | Interviewer/facilitator: Which author/s conducted the interview or focus group? | Pg 10 |
| 2 | Credentials: What were the researcher's credentials? e.g., PhD, MD | Pg 10 |
| 3 | Occupation: What was their occupation at the time of the study? | Pg 10 |
| 4 | Gender: Was the researcher male or female? | Pg 10 |
| 5 | Experience and training: What experience or training did the researcher have? | Pg 10 |
| **Relationship with participants** |  |  |
| 6 | Relationship established: Was a relationship established prior to study commencement? | Pg 10 |
| 7 | Participant knowledge of the interviewer: What did participants know about the researcher? (e.g., personal goals, reasons for doing the research) | Pg 10 |
| 8 | Interviewer characteristics: What characteristics were reported about the interviewer/facilitator? (e.g., bias, assumptions, reasons and interests in the research topic) | Pg 10-11 |
| **Domain 2: Study design** |  |  |
| **Theoretical Framework** |  |  |
| 9 | Methodological orientation and Theory: What methodological orientation was stated to underpin the study? (e.g., grounded theory, discourse analysis, ethnography, phenomenology, content analysis) | Pg 5-6 |
| **Participant Selection** |  |  |
| 10 | Sampling: How were participants selected? (e.g., purposive, convenience, consecutive, snowball) | Pg 7-8 |
| 11 | Method of approach: How were participants approached? (e.g., face-to-face, telephone, mail, email) | Pg 7-8 |
| 12 | Sample size: How many participants were in the study? | Pg 8 |
| 13 | Non-participation: How many people refused to participate or dropped out? Reasons? | Pg 9-10 |
| **Setting** |  |  |
| 14 | Setting of data collection: Where was the data collected? (e.g., home, clinic, workplace) | Pg 7 |
| 15 | Presence of non-participants: Was anyone else present besides the participants and researchers? | Pg 9-10 |
| 16 | Description of sample: What are the important characteristics of the sample? (e.g., demographic data, date) | Pg 8 |
| **Data Collection** |  |  |
| 17 | Interview guide: Were questions, prompts, guides provided by the authors? Was it pilot tested? | Pg 10 |
| 18 | Repeat interviews: Were repeat interviews carried out? If yes, how many? | N/A |
| 19 | Audio/visual recording: Did the research use audio or visual recording to collect the data? | Pg 10 |
| 20 | Field notes: Were field notes made during and/or after the interview or focus group? | Pg 9-10 |
| 21 | Duration: What was the duration of the interviews or focus group? | Pg 10 |
| 22 | Data saturation: Was data saturation discussed? | Pg 11-13 |
| 23 | Transcripts returned: Were transcripts returned to participants for comment and/or correction? | 12-13 |
| **Domain 3: Analysis and findings** |  |  |
| **Data analysis** |  |  |
| 24 | Number of data coders: How many data coders coded the data? | Pg 11-12 |
| 25 | Description of the coding tree: Did authors provide a description of the coding tree? | Pg 11-12 (partial) |
| 26 | Derivation of themes: Were themes identified in advance or derived from the data? | Pg 11-12 |
| 27 | Software: What software, if applicable, was used to manage the data? | Pg 11-12 |
| 28 | Participant checking: Did participants provide feedback on the findings? | Pg 12-13 |
| **Reporting** |  |  |
| 29 | Quotations presented: Were participant quotations presented to illustrate the themes/findings? Was each quotation identified (e.g., participant number)? | Pg 14-26 |
| 30 | Data and findings consistent: Was there consistency between the data presented and the findings? | Pg 14-26 |
| 31 | Clarity of major themes: Were major themes clearly presented in the findings? | Pg 14-26 |
| 32 | Clarity of minor themes: Is there a description of diverse cases or discussion of minor themes? | Pg 4-26 |

**Supplementary file 2: Field note/reflective journal sample**

Reflective journal entry 1: early impressions of Site A
Date: 3 March 2022
Location: Site A, ICU (AM shift)

Today was my first full observational day at Site A. I was struck by the openness of the nursing staff. Nurses appeared genuinely comfortable engaging with updated guidelines, laminated sheets were being used without prompting during ward rounds. The leadership presence was palpable: nurse managers were visible, accessible, and often facilitated quick policy huddles.

However, I noticed an interesting tension between formal evidence and intuitive practice. During a rapid deterioration event, a senior nurse reacted instinctively (correctly) before protocols could be consulted. It reminded me that while evidence is critical, experience and gut feeling are deeply woven into critical care practice. I need to explore this duality further in interviews.

Reflective journal entry 2: midway reflections on emerging patterns
Date: 12 May 2022
Location: Site B, CCU (Twilight shift)

Observing at Site B has been revealing but also challenging. Compared to Site A, there is a more hierarchical structure, senior physicians dominate decision-making, and nurses often defer without voicing concerns.

Interestingly, informal peer learning seems to fill the gaps where formal guideline updates are lacking. Conversations during breaks are rich with clinical 'tips' and workarounds. Yet, this also risks perpetuating outdated practices.

I’m increasingly aware of my positionality here. As an outsider and academic, I sense that some staff are cautious in my presence. I've been actively maintaining a "learner" stance, reiterating that I'm observing, not judging. This feels vital to building trust for authentic fieldwork.

Field Note 1 observation of knowledge dissemination at Site A
Date: 15 March 2022
Location: Site A, HDU

0830-1000 Morning shift huddle: NM1 led a structured 15-minute team briefing. Updated protocols for managing central line infections were discussed. Laminated A4 updates distributed.
Staff actively engaged, 2 nurses asked clarifying questions. Visible comfort with asking questions; NM1 encouraged it. Culture appears psychologically safe for knowledge-sharing.

Analytic memo: Leadership visibly operationalises EBP by normalising updates and reinforcing them in daily routines.

Field note 2: observation of informal knowledge sharing at site B
Date: 20 May 2022
Location: Site B, ICU

1415-1545 Afternoon shift: Observed SN5 and SN6 discussing an airway management case from the previous shift during a coffee break.
No formal guidelines referenced; knowledge exchanged based purely on memory of 'what worked before.' Staff mentioned they were “too busy” to look for the updated protocol online.

No evidence of posted reminders about guideline changes in the staff break room. Break rooms crowded; sense of exhaustion among nurses palpable.

Analytic memo: Time constraints and limited access to digital resources reinforce reliance on experiential knowledge over formal EBP.

**Supplementary file 3: Interview Guide**

| **Section** | **Sample Questions/Prompts** |
| --- | --- |
| **Opening/contextual** | Can you describe your role in this critical care unit?  What does a typical shift look like for you? |
| **Sources of knowledge** | During your shift, what types of information do you typically use when making clinical decisions?  How do you usually hear about new clinical guidelines or evidence updates? |
| **Knowledge sharing** | Can you describe how you usually share information or updates with your colleagues?  What formal or informal routines exist in your unit to support knowledge sharing? |
| **Guideline use** | How do you usually incorporate new knowledge or guideline updates into your practice?  What happens if a guideline contradicts what you have learned from experience? |
| **Experiential knowledge and intuition** | Can you share a time when your clinical intuition guided your decision-making?  How is experiential knowledge valued in your team? |
| **Organisational influences** | How does leadership in your unit support (or hinder) evidence-based practice?  How would you describe your team’s culture around using new evidence? |
| **Barriers and challenges** | What are the biggest challenges you face when trying to use the latest evidence in your practice?  Are there times when time pressures prevent you from accessing or using guidelines? |
| **Adaptive strategies** | How do you adapt when formal protocols don't quite fit the situation?  Can you describe how peer discussions help you integrate new knowledge into practice?" |
| **Closing** | Is there anything else you think would help improve knowledge use in critical care nursing?  Any other experiences you’d like to share? |

**Supplementary file 4: Coding framework**

| **Main Code** | **Sub-Codes** | **Description** | **Example from Data** |
| --- | --- | --- | --- |
| **Sources of knowledge** | Access to guidelines Peer learning Mentorship | Different ways nurses accessed and shared clinical knowledge. | "We have monthly governance meetings where updates are discussed." (A-NM2) |
| **Institutional influences** | Organisational culture Leadership engagement Hierarchy | Organisational structures and relationships that influenced knowledge use. | "We’re encouraged to speak up during ward rounds if something feels off." (A-ACP3) |
| **Experiential knowledge and intuition** | Clinical intuition Case memory Tacit learning | The use of prior experience and intuitive judgments in practice. | "Sometimes you just know something’s wrong even before the numbers change." (A-SN5) |
| **Barriers to EBP** | Time constraints Inconsistent dissemination Resistance to change | Factors that limited evidence-based practice use during clinical work. | "There’s no time to look up evidence when the ward is full." (B-ACP2) |
| **Strategies for integration** | Peer discussions Simulation-based learning Digital resources | How nurses bridged gaps between evidence and practice. | "After a major incident, we always have debriefs where we discuss what worked and what didn’t." (A-NM3) |
| **Contextual factors** | Staffing pressures Workforce shortages Technology access | Broader systemic and operational influences on knowledge application. | "Simulation is optional here, and digital access is poor during shifts." (Field notes, Site B) |

**Supplementary file 5: Audit Trail**

| **Stage** | **Description** | **Supporting Documents** |
| --- | --- | --- |
| **Pre-fieldwork preparation** | Research design developed; ethics approval obtained; hospital access negotiated; participant information sheets and consent forms prepared. | Ethics approval letter, Hospital permission letters, Study Protocol |
| **Field entry** | Initial meetings with unit managers; observer role explained to staff; Observer badges worn; rapport building commenced; non-participant observer status maintained. | Field access emails, Researcher field notes |
| **Data collection** | 56 non-participant observation sessions, 36 semi-structured interviews, and document reviews conducted over eight months. Reflective journals kept throughout field engagement. | Observation logs, Interview transcripts, Reflective memos, Document analysis logs |
| **Data management** | Audio-recordings transcribed verbatim; field notes typed and organised; data uploaded to NVivo 12; systematic coding frameworks developed. | Transcripts, NVivo project files, Coding manual |
| **Initial data analysis** | Spradley’s domain and taxonomic analysis performed iteratively; field notes triangulated with interviews and documents; preliminary domains identified. | Domain analysis tables, Reflexive journal entries |
| **Cross-coding and peer debriefing** | 25% of transcripts and fieldnotes cross-coded by third author; discrepancies discussed and resolved through analytic team meetings. | Cross-coding spreadsheets, Meeting minutes |
| **Theme development** | Themes refined through iterative coding cycles; thematic synthesis mapped against research aims; member checking undertaken via participant feedback on anonymised theme summaries. | Thematic matrices, Member checking summaries |
| **Interpretation and reporting** | Themes interpreted in relation to research questions, theoretical framework, and existing literature. Findings organised by major and minor themes; direct participant quotes incorporated. | Draft findings chapter, Peer review feedback notes |
| **Final verification** | Final audit trail reviewed to ensure transparency; all documents systematically archived; reflexive commentary finalised. | Final reflexive journal, Data archive inventory |

**Supplementary file 6: Member checking summary**

| **Stage** | **approach** | **feedback from participants** | **action taken** |
| --- | --- | --- | --- |
| **Theme development stage** | Participants provided anonymised summaries of emergent themes after preliminary analysis. | Participants confirmed the resonance of major themes (e.g., leadership, time constraints, peer learning). Some requested clearer representation of the use of intuition and experiential learning. | Incorporated more explicit subthemes on clinical intuition and experiential case memory within main themes. |
| **Cross-site comparison** | Participants from both sites were asked whether site-specific differences accurately reflected their experiences. | Site A participants agreed with portrayal of supportive culture. Site B participants confirmed hierarchical influences and sporadic updates but requested a less negative tone. | Adjusted language to reflect systemic challenges rather than blame individuals. Highlighted resource-related barriers more explicitly. |
| **Validation of final themes** | Selected participants were invited to comment on the final thematic map and framework. | Strong agreement overall. Participants emphasised the importance of showing how informal learning (e.g., peer discussions) functioned as a strength, not just a gap. | Enhanced findings narrative to highlight peer discussions as a crucial strategy for navigating knowledge barriers. |
| **Reflexive feedback** | During feedback sessions, participants noted that pandemic-related pressures may have amplified some barriers during the study period. | Added caveats in the discussion regarding COVID-19's potential amplifying effect on workload and knowledge dissemination. |  |
